# *n*-Butyl-2-cyanoacrylate tissue adhesive (Histoacryl) vs. subcuticular sutures for skin closure of Pfannenstiel incisions following cesarean delivery

**DOI:** 10.1371/journal.pone.0202074

**Published:** 2018-09-14

**Authors:** Ji Young Kwon, Hang Goo Yun, In Yang Park

**Affiliations:** Department of Obstetrics and Gynecology, The Catholic University of Korea, Seoul, Korea; Medical University Graz, AUSTRIA

## Abstract

**Background:**

Tissue adhesives are now routinely used for skin closure in various surgeries. This study aimed to evaluate the safety and efficacy of *n*-butyl-2-cyanoacrylate (NBCA) tissue adhesive in cesarean delivery by comparing it with the safety and efficacy of subcuticular suture closure.

**Methods and findings:**

A retrospective chart review was undertaken of all patients who underwent cesarean delivery via Pfannenstiel skin incision. During the study period, a total of 209 patients had NBCA (Histoacryl^®^) closure and 208 patients had suture closure. Wound complications and Vancouver scar scale (VSS) scores were compared between the 2 groups.

**Results:**

There were no significant differences between the two groups in indications for cesarean deliveries or number of previous cesarean deliveries. Incidences of wound disruption and infection were also similar between the two closure groups (*p* = 0.322 and 0.997, respectively). The rate of wound complications was 3.4% in the NBCA group and 5.3% in the suture group. All complications healed uneventfully with topical antibiotics or closure strips. VSS scores at 6–8 weeks after operation were not significantly different between the two groups (*p* = 0.858). These results were corroborated by propensity score-matching analysis.

**Conclusions:**

NBCA may be a useful skin closure of Pfannenstiel skin incisions after cesarean delivery.

## Introduction

Cesarean delivery is one of the most commonly performed surgeries worldwide. About 26% of pregnant women in OECD countries deliver by cesarean [1]. Therefore, efforts to reduce cesarean wound complications are necessary not only for patients, but also for national public health management. Cesarean delivery requires a relatively long skin incision, and efficient healing of the cesarean wound is a very important determinant of the postoperative satisfaction of the patient.

The skin wound from cesarean section is generally closed with surgical staples or sutures [2–4]. Although staples have the advantage of reducing operative time, they are likely to cause more wound disruption or infection than suture closure [3]. Suture closure is a safe and effective method, but time consuming and operator dependent, and there is a risk of needle stick injury. Furthermore, an additional procedure is required to remove the sutures, and the timing of suture removal affects the degree of scarring [4]. Rapid removal of the suture can result in weak wound tensile strength, resulting in a widened scar, while late removal can result in scar formation due to inflammation [5].

*n*-butyl-2-cyanoacrylate (NBCA) is a tissue adhesive that has been used as an alternative to sutures for more than 40 years. NBCA in liquid solution is monomeric; application to tissue results in polymerization, resulting in a strong tissue bond that holds the wound edges together [6,7]. Tissue adhesive is easier and faster to apply than suture closure and it forms a waterproof and bactericidal barrier. It does not require removal because the wound is spontaneously sloughed off after re-epithelization. Furthermore, some studies have suggested that it may improve cosmetic outcome [6–9]. Because of these advantages, NBCA is now used for skin closure in various surgeries [9–13], but its use in cesarean skin wounds is not yet common and a few studies have assessed the feasibility of using a tissue adhesive for skin closure of cesarean Pfannenstiel incisions [14, 15].

Therefore, in this study, we compared the wound outcomes of NBCA tissue adhesive for Pfannenstiel skin closures following cesarean delivery compared with subcuticular suture closure.

## Materials and methods

A retrospective chart review was undertaken of all patients who underwent cesarean delivery via Pfannenstiel skin incision at a university hospital in Korea from January 2015 and August 2016. All cesarean types were included; scheduled or emergency and primary or repeat. During this period, three obstetricians were responsible for all deliveries. The skin incision was closed by NBCA tissue adhesive (Histoacryl^®^; B. Braun, AG, Melsungen, Germany) or suture, and the decision on which method to use for skin closure was based on the operator’s preference. Skin closure was performed by one of three obstetric clinical fellows. During the study period, a total of 209 patients had NBCA closure and 208 patients had suture closure. This study was approved by the institutional Review Board (IRB) of the Catholic University of Korea (reference No. KC16RISI0551). The patient records were accessed anonymously and the IRB waived the need for informed consent. The patient records were accessed anonymously and the IRB waived the need for informed consent. All the researchers did not have any conflict of interest to declare.

**Fig 1:**
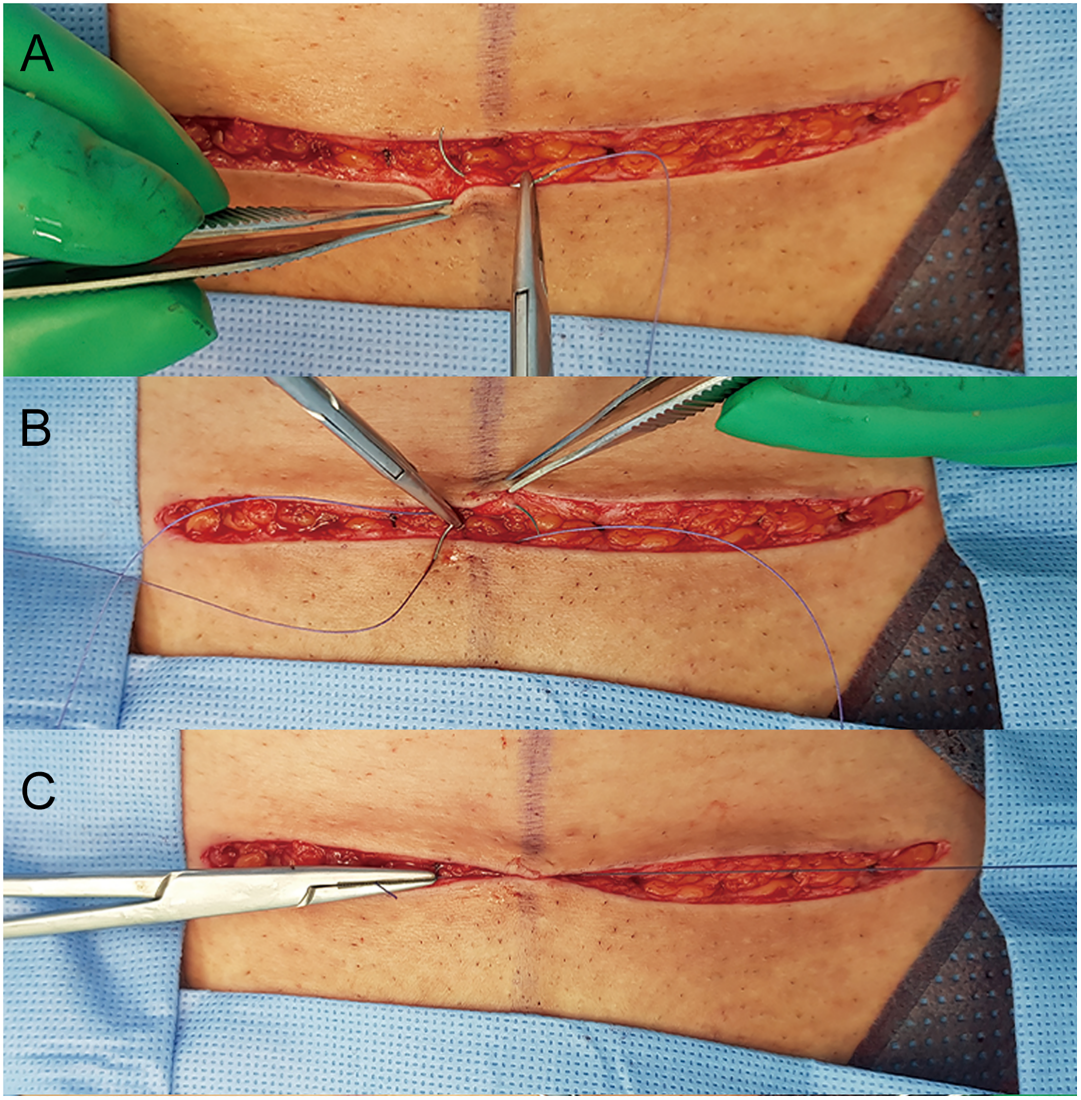
To achieve an even distribution of tension, the wound edges were approximated with interrupt buried intradermal sutures at 1 cm intervals of 3–0 Vicryl (A-C).

**Fig 2:**
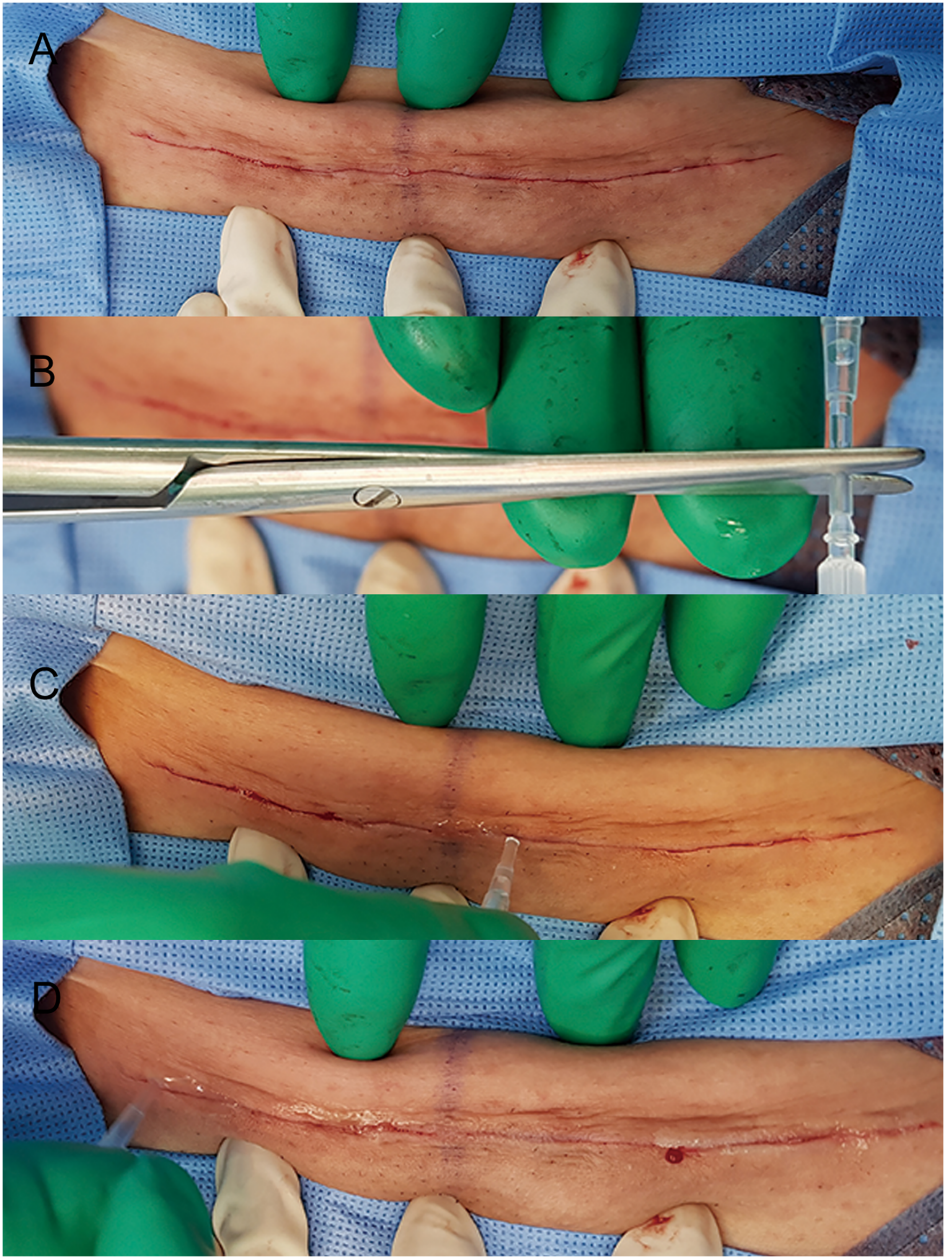
In cases of tissue adhesive closure, skin edges was manually approximated (A). Histoacryl was opened by cutting the tip of cannula and activated (B). Histoacryl was applied over approximated wound edges as a thin film. (C, D).

The cesarean technique followed usual practices including perioperative prophylactic antibiotics. Per protocol, 1 g of cephoxitin was administrated. For women with penicillin or cephalosporin allergy, 900 mg of clindamycin was given. In cases of repeat cesarean section, the previous cesarean scar was excised and the same site was incised. Closure of the fascia was performed using 1–0 Vicryl (Ethicon, Somerville, NJ, USA). Closure of subcutaneous fat was performed in women with fat thickness greater than 2 cm by standard methods using 2–0 Vicryl (Ethicon, Somerville, NJ, USA). To achieve an even distribution of tension, the wound edges were approximated with interrupt buried intradermal sutures at 1-cm intervals of 3–0 Vicryl (Fig 1). Next, for cases of NBCA skin closure, one layer of Histoacryl was applied over the approximated wound edges. Before applying tissue adhesive, the incision site was cleaned with sterile saline solution and dried with a gauze. And then, the ample of the Histoacryl was opened by cutting the tip of the cannula and the adhesive was applied over the manually approximated wound edges as a thin film. After applying adhesive, light pressure along the wound line should be maintained approximately manually for 30 seconds to achieve a full strength (Fig 2). A total of one vial of Histoacryl (0.5 ml) was used for each patient. No dressing was used and patients were permitted to shower the day after surgery. For cases of suture closure, the skin was closed with a running subcuticular suture with 4–0 Prolene. A standard absorbent dressing was applied and changed on the second day after operation. Patients returned to the hospital for suture removal on postoperative day 7–10 and adhesive closure strips (Steri-Strips^™^ 12 x 100 mm; 3M Health Care St, Paul, MN) were applied to the incisions. Showering was allowed after suture removal.

Data regarding wound complications were collected at the time of discharge from hospital records, and at the 1–2 weeks and 6–8 weeks postpartum visits. Wound disruption was considered minor when it was reapproximated with adhesive closure strips. When it required suture or staples, wound disruption was considered major. Wound infection was defined as purulent drainage, cellulitis, and abscess requiring antibiotics. The scar had been routinely assessed at 6–8 weeks postpartum by obstetricians using the Vancouver scar scale (VSS). The VSS is a 13-point scoring system consisted of four variables: vascularity, pigmentation, pliability, and height (thickness) [16]. Each variable has four to six possible scores, and a score of 0 means normal. The total of these scores ranges from 0 to 13, and a higher score indicates a scar with a poorer appearance (Table 1). Data on maternal baseline characteristics as well as wound outcomes were collected by a researcher who were not involved in the operation and scar assessment from chart review.

**Table 1:** Vancouver scar scale.

Parameter	Descriptor	Scores
Vascularity	Normal	0
	Pink	1
	Red	2
	Purple	3
Pigmentation	Normal	0
	Hypo-pigmentation	1
	Mixed-pigmentation	2
	Hyper-pigmentation	3
Pliability	Normal	0
	Supple (flexible with minimal resistance)	1
	Yielding (giving way to pressure)	2
	Firm (inflexible, not easily moved, resistant to manual pressure)	3
	Banding (rope-like tissue that blanches with extension of the scar)	4
	Contracture (permanent shortening of scar, producing deformity)	5
Height	Normal	0
	< 2 mm	1
	2–5 mm	2
	≥ 5 mm	3
Total score		13

### Statistical analysis

Baseline characteristics, wound complications, and VSS scores were compared between the NBCA and suture groups. And then, the differences in baseline characteristics of patients were adjusted using propensity score. We calculated propensity scores for each patient, using logistic regression analysis involving the following factors; age, BMI at pre-pregnancy, weight at delivery, the presence or absence of diabetics, hypertensive disease, and immunologic disease, the number of fetus, and history of previous cesarean delivery. One-to-one propensity score matching was performed by the Matchlt matching method using a macro. Categorical variables were compared using the Chi-square test or Fisher’s exact test and continuous variables were compared between groups using Student’s t-test or the Wilcoxon rank-sum test. Also, we performed a subgroup analysis for obese women with a BMI of 25 or greater. A *p* value < 0.05 was considered statistically significant. SPSS (version 23.0; SPSS Inc., Chicago, IL, USA) was used for statistical analyses.

## Results

There were no differences in baseline characteristics between the two groups including maternal age, parity, pre-pregnancy weight, body mass index, and medical comorbidities such as hypertensive diseases, diabetic status, and presence of immunologic disorders. Indications for cesarean section and number of previous cesarean deliveries were also not significantly different between the two groups (Table 2).

**Table 2:** Maternal characteristics and pregnancy outcomes.

Variables	Entire Cohort (N = 417)			Propensity Score-Matched Cohort (N = 356)		
	Tissue adhesive (N = 209)	Suture (N = 208)	*p* value	Tissue adhesive (N = 178)	Suture (N = 178)	*p* value
Maternal age (years)	33.9 ± 3.8	34.6 ± 4.0	0.083	34.1 ± 3.7	34.3± 3.9	0.791
Multipara	88 (48.9)	92 (51.1)	0.693	75 (42.1)	80 (44.9)	0.669
Height (cm)	161.6 ± 0.1	162.0 ± 0.1	0.435	161.7 ± 0.1	161.5 ± 0.1	0.728
Pre-pregnancy weight (kg)	56.0 ± 9.1	56.8 ± 10.0	0.403	55.8 ± 8.8	56.8 ± 10.3	0.344
Weight at delivery (kg)	67.8 ± 10.1	69.6 ± 11.4	0.079	67.8 ± 10.1	69.3 ± 11.2	0.191
Pre-pregnancy BMI (kg/m^2^)	21.5 ± 3.3	21.7 ± 3.5	0.601	21.3 ± 3.1	21.7 ± 3.6	0.257
BMI ≥ 25 kg/m^2^	24 (11.5%)	31 (14.9%)	0.187	16 (9.0%)	26 (14.6%)	0.139
Previous cesarean delivery (No.)			0.562			0.686
1	65(31.1)	59 (28.6)		56 (31.5)	52 (29.2)	
≥ 2	7 (3.3)	11 (5.4)		6 (3.4)	9 (5.1)	
Hypertensive disorder*	14 (6.7)	13 (6.3)	1.000	9 (5.1)	12 (6.7)	0.654
Diabetics	13 (6.2)	12 (5.8)	1.000	10 (5.6)	11 (6.2)	1.000
Immunologic disorder	2 (1.0)	1 (0.5)	0.565	0 (0.0)	1 (0.6)	0.317
Gestational age (week)	36.3 ± 3.1	36.9 ± 3.6	0.096	36.5 ± 3.0	36.9 ± 3.7	0.316
Preterm delivery (< 32 weeks)	26 (12.4%)	28 (13.5%)	0.773	19 (10.7%)	26 (14.6%)	0.339
Multifetal pregnancy	3 (1.5)	4 (1.9)	0.698	3 (1.7)	2 (1.1)	0.652
Indication of cesarean			0.197			0.129
Elective	168 (80.4)	155 (75.2)		144 (80.9)	131 (73.6)	
Emergency	41 (19.6)	53 (25.5)		34 (19.1)	47 (26.4)	

Data are expressed as number (%) or mean ± standard deviation

BMI, Body mass index

*Pregestational, gestational, and preeclampsia

Cumulative incidences of wound complications at the time of discharge, at 1–2 weeks, and at 6–8 weeks were similar in the two closure groups (Table 3). There was no significant difference in wound complications. The overall complication rate was 3.4% in the NBCA group and 5.3% in the suture group. Wound disruption occurred in six patients (2.9%) in the NBCA group and 10 patients (4.8%) in the suture group. These wounds healed uneventfully with adhesive closure strips. There was no incidence of wound dehiscence requiring a suture in either group. Wound infection occurred in one patient in each group. These wound infections were treated with topical and oral antibiotics. There were no allergic reactions associated with NBCA.

**Table 3:** Wound complications by skin closure.

Variables	Entire Cohort			Propensity Score-Matched Cohort		
	Tissue adhesive (N = 209)	Suture (N = 208)	*p* value	Tissue adhesive (N = 178)	Suture (N = 178)	*p* value
Wound disruption	6 (2.9)	10 (4.8)	0.322	4 (2.2)	8 (4.5)	0.240
<1 cm	2 (1.0)	4 (1.9)		0 (0.0)	3 (1.7)	
1–2 cm	4 (1.9)	6 (2.9)		4 (2.2)	5 (2.8)	
Wound infection	1 (0.5)	1 (0.5)	0.997	1 (0.6)	1 (0.6)	1.000

Data are expressed as number (%)

Cosmetic evaluation was available in 371 patients from chart review. There was no significant difference in VSS scores between the two skin closure groups (Table 4). Median total VSS score was 1 in both the NBCA and suture groups at 6–8 weeks after the operation.

**Table 4:** Score of the Vancouver scar scale at 6–8 weeks postoperatively.

Variable	Entire Cohort			Propensity Score-Matched Cohort		
	Tissue adhesive (N = 191)	Suture (N = 180)	*p* value	Tissue adhesive (N = 163)	Suture (N = 153)	*p* value
Vascularization	0 (0–1) 0–2	0 (0–1) 0–2	0.541	0 (0–1) 0–2	0 (0–1) 0–2	0.900
Pigmentation	0 (0–1) 0–2	0 (0–1) 0–2	0.540	0 (0–1) 0–2	0 (0–1) 0–2	0.630
Pliability	1 (0–1) 0–4	1 (0–1) 0–4	0.923	1 (0–1) 0–4	1 (0–1) 0–4	0.766
Height	0 (0–0) 0–2	0 (0–0) 0–3	0.842	0 (0–0) 0–2	0 (0–0) 0–3	0.793
Total score*	1(1–2) 0–8	1(1–2) 0–9	0.858	1(1–2) 0–8	1 (1–2) 0–9	0.696

Data are medina (interquartile range) and range.

* 0 = best possible score; 13 = lowest possible score

To confirm the validity of the tissue adhesive for skin closure, we conducted one-to-one propensity score-matched analysis. Pairs of 178 patients were obtained and well matched for baseline characteristics (Table 2). Wound complications were observed in 14 of 356 patients in the propensity score-matched cohort. In this cohort, the incidence of wound complications showed no significant difference between two methods of skin closure (Table 3). Also, Cosmetic evaluation using VSS scores showed no significant differences between two skin closure groups in the propensity score-matched cohort (Table 4).

In sub-analysis for women with a BMI of 25 or greater, there were no significant difference in wound complication between two groups (*p* = 0.436). Only one of 24 patients (4.2%) in the NBCA group had wound disruption and it was wound disruption less than 1 cm. and none in the suture group had wound complication. Comparison of VSS scores showed no differences between two method (*p* = 0.503, 0.752, 0.144, and 0.916 for vascularization, pigmentation, pliability, and height, respectively).

## Discussion

The main finding of this study is that NBCA tissue adhesive can be safely and effectively used for skin closure after cesarean delivery. Tissue adhesive for skin closure is now being used to close many surgical wounds as an alternative to sutures. However, it is rarely used in cesarean section, one of the most common surgeries in the world. This may be due to the lack of clinical experience with tissue adhesive application for skin closure of Pfannenstiel incisions after cesarean delivery. This study suggests that use of NBCA results in wound outcomes equivalent to those of sutures for Pfannenstiel incisions, indicating that NBCA is a safe option for cesarean skin closure.

The present study was conducted on consecutive pregnant women who underwent cesarean delivery via Pfannenstiel skin incision. Therefore, it includes all type of cesarean section; scheduled or unscheduled and primary or repeat. Additionally, pregnant women with risk factors for delayed wound healing such as obesity, diabetes, and immune diseases were included. And, Although we did not use a regression model to analyze the outcomes of using NBCA under these specific conditions because there were so few cases of wound complications, there do not appear to be specific obstetric characteristics or medical conditions in which NBCA skin closure should be avoided. However, NBCA should not be used in patients with allergies to cyanoacrylate, formaldehyde, tapes, or adhesives.

Previous studies to suggest the efficacy of tissue adhesive comparable to convetional suture have focused on the skin closure of small incisional surgical wound such as laparoscopic port site or thyroidecomty [17,18]. Using tissue adhesives in major surgeries involving long skin incisions was recently founded to be safe in breast surgery and abdominoplasty [19,20]. Additionally, one recent randomized study suggested that the use of tissue adhesive at cesarean section had similar wound complication and scar assessment scale compared with suture method [15]. This is similar results to our study, which indicate that NBCA closure of Pfannenstiel skin incisions is both feasible and safe.

The primary endpoint of surgical site closure is wound healing without complications, and the secondary endpoint is cosmetic outcome. In this study, wound complications such as wound disruption or infection occurred in seven cases (3.4%) among 209 patients with NBCA skin closure. This incidence was not significantly different from that of suture closure. Furthermore, no cases required re-suturing, and minor complications were successfully treated with adhesive closure strips and topical antibiotics. The median total VSS score, which was evaluated at 6–8 weeks of operation, was 1, indicating that cosmetic outcome after NCBA closure was excellent and not statistically different from that of suture closure. Because hypertrophic scar formation occurs within 3 months after surgery [21], it is more accurate to evaluate cosmetic outcomes at least 3 months after the operation. However, given that scar formation is occurring at this time, the excellent cosmetic outcome achieved after NCBA closure is meaningful. Many studies have reported that tissue adhesives resulted in the same or better cosmetic outcomes and no increase in wound complications compared to suture closure [15,17–20].

When wound outcome is equivalent, selection of the wound closure method depends on the preference of the patient or the surgeon. Tissue adhesive is a very attractive choice for both the patient and surgeon. NCBA has the great advantage of not requiring removal of sutures after surgery. Although removal of sutures is not a painful or time-consuming process, patients may be anxious about this remaining procedure. For patients who live in remote locations, it is also inconvenient to have to return to the hospital. Furthermore, if suture removal is delayed or performed a few days early because of the patient’s and/or physician’s schedules, the risk of wound complications or scar formation increases. Tissue adhesive does not require a bandage for wound cover, and the patient can shower immediately after surgery. Sutures need to be covered to maintain waterproof and aseptic conditions in the wound. The bandage may cause skin irritation and need to be changed, which is not only inconvenient but also imposes additional costs. For doctors, the advantage of tissue adhesive is a reduction in operating time. Three randomized controlled trials reported that wound closure using tissue adhesive takes a significantly shorter time than wound closure by suture [22–24]. This operative time saving may be greater in surgeries with long incision sites. In the case of abdominoplasty, which involves a long incision of 20 cm or more, skin closure using tissue adhesive rather than suture reduced the operation time by 13 minutes on average [20]. We did not analyze the time it took for skin closure. However, because a long incision is made in cesarean section, the total operating time is likely to be significantly lower with tissue adhesive skin closure than suture skin closure.

We did not perform cost analysis of tissue adhesive closure and suture closure. In almost all countries, tissue adhesive is a more expensive material than suture material. However, cost analysis requires consideration of operating room time, wound bandage costs, and costs of the removal of sutures. A previous studies that performed a cost analysis reported that tissue adhesive closure in laparoscopic-port site, abdominoplasty surgery, and breast surgery resulted in cost savings compared to suture closure [20,24].

This study had some limitations. Due to its retrospective study, confounding variables may have been present. However, to minimize potential bias, we investigated consecutive cases of all cesarean deliveries via Pfannenstiel skin incision, and there were no significant differences between the baseline characteristics of the two groups. Also, we confirmed the similar results by propensity score-matching analysis. Furthermore, data surveys were conducted by a researcher who was not involved in surgery and scar assessment. Skin closure was performed by one of four obstetric fellows, all of whom were skilled in wound suture and Histoacryl application. Although cosmetic outcomes of all included patients were not analyzed, the number of patients lost to follow-up was similar in the two groups. Additionally, the study cohort had relatively low mean BMI and women with BMI of 25 kg/m2 or more was only 13.2% of the cohort, which is lower than that for other countries. Generally, about 60% of Korean women start their pregnancies with optimal BMI, 18.5–23 kg/m^2^ by Korean BMI categories. Further studies for obese women is needed to use NBCA regardless of BMI.

## Conclusion

In conclusion, our results indicate that NCBA tissue adhesive may be useful for skin closure of Pfannenstiel skin incisions after cesarean delivery. Despite their many advantages, tissue adhesives have not been widely used for skin closure in cesarean section. NCBA skin closure in cesarean delivery can achieve favorable cosmetic results with no increases in wound complication rates. Well-designed controlled studies that examine patient satisfaction, long-term cosmetic outcomes, and operating times should be performed in the future.

## Supporting information

S1 File Data on maternal baseline characteristics and wound outcomes for all included patients. (XLSX)
